# Association of Anti-Rotavirus IgA Seroconversion with Growth, Environmental Enteric Dysfunction and Enteropathogens in Rural Pakistani Infants

**DOI:** 10.1016/j.vaccine.2022.04.032

**Published:** 2022-05-31

**Authors:** Sheraz Ahmed, Junaid Iqbal, Kamran Sadiq, Fayaz Umrani, Arjumand Rizvi, Furqan Kabir, Zehra Jamil, Sana Syed, Lubaina Ehsan, Fatima Zulqarnain, Muhammed Sajid, Aneeta Hotwani, Najeeb Rahman, Jennie Z. Ma, Monica McNeal, Sue Ann Costa Clemens, Najeeha Talat Iqbal, Sean R. Moore, Asad Ali

**Affiliations:** a Department of Pediatrics and Child Health, Aga Khan University, Karachi, Pakistan; b Department of Biological and Biomedical Sciences, Aga Khan University, Karachi, Pakistan; c Division of Pediatric Gastroenterology, Hepatology, and Nutrition, Department of Pediatrics, University of Virginia, Charlottesville, USA; d Department of Public Health Sciences, University of Virginia, Charlottesville, USA; e Division of Infectious Diseases, Department of Pediatrics, University of Cincinnati College of Medicine, OH, USA; f Institute for Global Health, University of Siena, Italy

**Keywords:** Oral vaccines, Infants, Stunting, Biomarkers, Environmental enteric dysfunction, Malnutrition, Rotavirus vaccine

## Abstract

**Background:**

The underperformance of oral vaccines in children of low- and middle-income countries is partly attributable to underlying environmental enteric dysfunction (EED).

**Methodology:**

We conducted a longitudinal, community-based study to evaluate the association of oral rotavirus vaccine (Rotarix®) seroconversion with growth anthropometrics, EED biomarkers and intestinal enteropathogens in Pakistani infants. Children were enrolled between three to six months of their age based on their nutritional status. We measured serum anti-rotavirus immunoglobulin A (IgA) at enrollment and nine months of age with EED biomarkers and intestinal enteropathogens.

**Results:**

A total of 391 infants received two doses of rotavirus (RV) vaccine. 331/391 provided paired blood samples. Of these 331 children, 45% seroconverted at 9 months of age, 35% did not seroconvert and 20% were seropositive at baseline. Non-seroconverted children were more likely to be stunted, wasted and underweight at enrollment. In univariate analysis, insulin-like growth factor (IGF) concentration at 6 months were higher in seroconverters, median (25th, 75th percentile): 26.3 (16.5, 43.5) ng/ml vs. 22.5 (13.6, 36.3) ng/ml for non-seroconverters, p-value = 0.024. At nine months, fecal myeloperoxidase (MPO) concentrations were significantly lower in seroconverters, 3050(1250, 7587) ng/ml vs. 4623.3 (2189, 11650) ng/ml in non-seroconverted children, p-value = 0.017. In multivariable logistic regression analysis, alpha-1 acid glycoprotein (AGP) and IGF-1 concentrations were positively associated with seroconversion at six months. The presence of sapovirus and rotavirus in fecal samples at the time of rotavirus administration, was associated with non-seroconversion and seroconversion, respectively.

**Conclusion:**

We detected high baseline RV seropositivity and impaired RV vaccine immunogenicity in this high-risk group of children. Healthy growth, serum IGF-1 and AGP, and fecal shedding of rotavirus were positively associated with RV IgA seroconversion following immunization, whereas the presence of sapovirus was more common in non-seroconverters.

**Trial registration**: Clinical Trials ID: NCT03588013.

## 1 Background

Environmental Enteric Dysfunction (EED) is a subclinical disorder characterized by impaired gut immune function, malabsorption, growth faltering, increased intestinal permeability, and oral vaccine failure [1].Histologically, EED is characterized by villous atrophy, abnormal crypts to villous ratio, and an increased number of lymphocytes and plasmacytes in the lamina propria [2]. Oral vaccine performance can be influenced by alterations in gut structure, which may affect the absorption of the vaccine [3]. In general, all live oral vaccines (both viral and bacterial) are less efficacious in LMICs [4], [5], [6]. In addition to compromised gut health, other hypothesized reasons include the maternal transfer of antibodies to the infant [7], EED [8], enteric pathogens [9], interference of breast milk antibodies against vaccine antigens [10], malnutrition and micronutrient deficiencies [11] and histo-blood groups antigens [12], [13], [14]. However, current evidence on interrelationship between EED and oral vaccines performance is not conclusive.

In 2013, Rotavirus (RV) globally contributed to an estimated 37% of all-cause diarrheal deaths in children under five years of age. RV was responsible for 33% of severe gastroenteritis in hospitalized children. Four countries (India, Pakistan, Nigeria, and DRC) accounted for 49% of all rotavirus deaths globally in this age group [15]. RV was estimated to be responsible for 258 million diarrheal episodes in children under five [16]and is the number one cause of diarrhea in Pakistani infants [17].

Currently, four anti-RV vaccines are WHO prequalified for global marketing for the prevention of RV infection [18], [19], [20], [21], [22]. There is evidence of anti-RV vaccine effectiveness of > 90% in developed countries [23] but low effectiveness of < 50% in low and middle-Income countries (LMIC) [4], [5], [6]. There is also evidence for the effect of seasonal variation, different rotavirus strains circulating in Asia and Africa, age at vaccine administration, and co-administration with other oral vaccines that could cause a decrease in oral vaccine efficacy and immunogenicity [24].

In order to explore for putative EED biomarkers, numerous studies have been conducted on gut-specific and systematic inflammatory biomarkers. Studies exploring the effectiveness of oral vaccines have assessed these markers in addition to seroconversion in order to gain a better insight [25], [26], [27], [28].

Seroconversion represents a specific antibody response in the blood (usually within a few weeks of either infection or immunization)[22]. Although it is an imperfect correlate of protection, serum anti-rotavirus IgA is widely utilized in immunogenicity trials as a measure of vaccine take. In this study, we attempted to understand correlations of RV vaccine IgA antibody seroconversion (vaccine take) with children’s growth, enteropathogens burden and EED biomarkers.

## 2 Methodology

### 2.1 Study population and objective

Data for this manuscript came from the enrolled children who were recruited in the Study of Environmental Enteropathy in Malnutrition (SEEM), a community-based interventional longitudinal trial in rural Pakistan between 2016 and 2019.The detailed methodology of SEEM has been described in protocol paper [29].

### 2.2 Data and lab samples collection

**Fig. 1:**
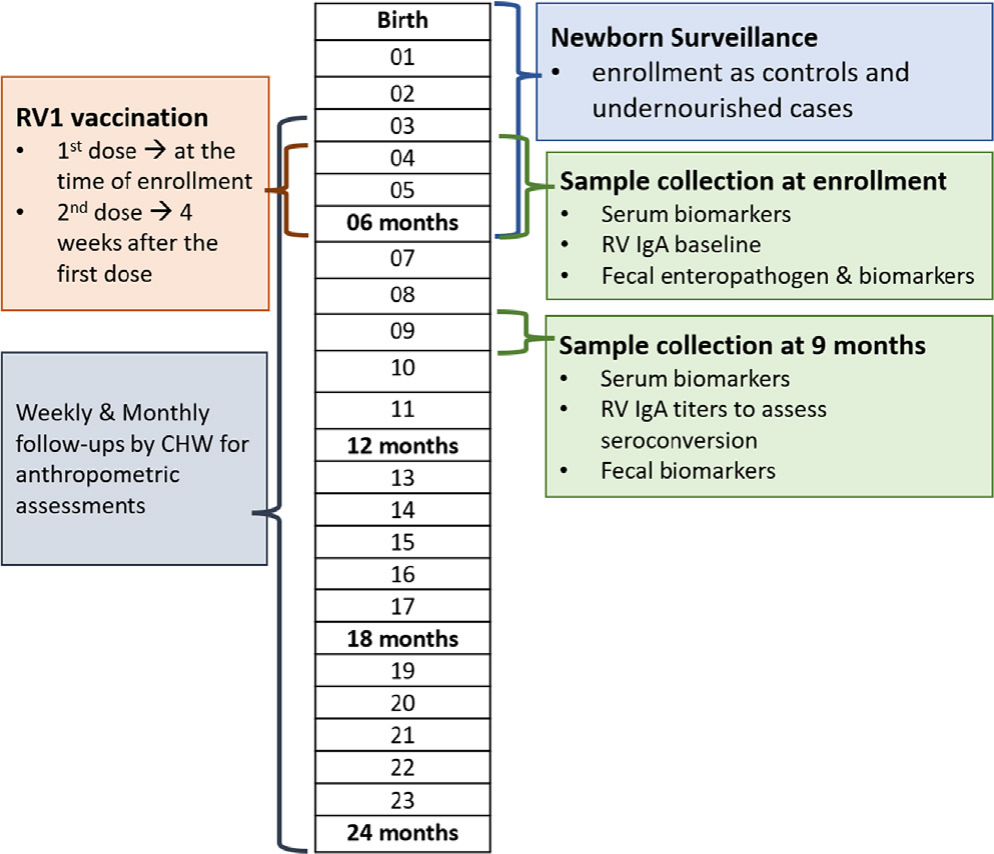
Methodological summary with timelines of study enrollment, anthropometric evaluation, vaccine administration and sample collection in the “Study of Environmental Enteropathy and Malnutrition” (SEEM).

We enrolled acutely malnourished children from 3 to 6 months of age if their Weight for Length Z score (WLZ score) was less than −2.0. Well-nourished children were enrolled based on WLZ ≥ 0 and Length for Height Z score (LAZ > -1) on two consecutive visits between 3 and 6 months of age. Exclusion criteria were parental refusal to consent or if the child was suffering from any congenital anomaly. Based on the anthropometric measurements and WHO growth standards, children were classified into the status of stunted, wasted, and/or underweight, each at the level of normal, moderate, and severe [30]at the time of enrollment. Blood and stool, samples were collected from enrolled children at 3–6 and at 9 months of age (Fig. 1**)**. Gut-specific biomarkers collected in this study were stool myeloperoxidase (MPO), and neopterin. Systematic inflammatory biomarkers were glucagon-like peptide 2 (GLP2), ferritin, C-reactive protein (CRP), leptin, pre-albumin, insulin-like growth factor (IGF) and α1-acid glycoprotein (AGP).

For targeted analyses of viral, bacterial and parasite load in the fecal samples, non-diarrheal stool was collected from the children at the time of enrollment using a stool kit provided to the parents of the study participants. All specimens were transported at 4 °C from field site lab to Karachi under cold chain maintenance. TaqMan Array Card (TAC) by Roche Diagnostics International AG, Rotkreuz, Switzerland, assay was used to detect quantitative estimates of both RNA and DNA (Total DNA) through real-time PCR. The sample was considered valid positive if 1) the sample’s target Ct value was<35.0 while the internal controls had a Ct value<35.0. These TAC cards were customized to detect 27 common enteropathogens such as giardia, ETEC, EPEC, EAEC, Campylobacter spp, Cryptosporidium spp. and common viruses associated with diarrhea in childhood [31].

### 2.3 Rotavirus vaccine administration

Rotavirus vaccine was procured through Aga Khan University pharmacy, transported to a field office at 2–8 °C, and stored in a field laboratory refrigerator under the recommended temperature. All enrolled children received oral, live monovalent rotavirus vaccine (RV1), RotaRix® (manufactured by the GSK Biologicals, Rixensart, Belgium) for the prevention of rotavirus gastroenteritis caused by G1 and non-G1 types (G3, G4, and G9)[32]. When we started this study, rotavirus vaccine was not included in the national immunization schedule and this vaccine was only available in some private hospitals. The purpose of vaccinating the enrolled children (majority of them were malnourished) was that we wanted to offer the children with some added benefit. Children received the first vaccine dose before the blood collection at time of enrollment. The second vaccine dose was administered 4 weeks after the first dose. Serum samples to measure rotavirus IgA antibodies were shipped to Cincinnati Children’s Hospital Medical Center, USA, for analysis. Serum rotavirus IgA antibody was measured by enzyme-linked immunosorbent assay (ELISA) as arbitrary Units/mL (AU) of serum as previously described [33].

### 2.4 Statistical analysis

The analysis was carried out in STATA version 16 SE (College Station, TX, USA) [34]. The malnourished and well-nourished children were combined as the seroconversion rate was not different between these two groups. Seroconversion was defined as a concentration of anti-rotavirus IgA antibodies ≥ 20 Arbitrary Units (AU)/mL in seronegative children (IgA < 20 U/mL) at the time of first blood sample collection [35]. Descriptive statistics were reported as frequency percent, mean/median with standard deviation and interquartile range (IQR) as appropriate. Independent two-sample *T*-test and Mann Whitney *U* test was used to test the difference in the continuous measures between seroconverted and non-seroconverted children. Spearman’s correlation coefficient was used to quantify the relationship between biomarkers concentration and IgA units. Logistic regression analysis was performed to assess the children’s characteristics and biomarkers associated with seroconversion. The covariates were child age, place of birth, gender, mother education, breastfeeding history, household food security index, nutritional indices before administration of the Rota vaccine and the change in biomarkers concentration between 6 and 9 months. Due to their skewed distribution, change in biomarker concentration were divided into four quartiles as Q1 (0–25th percentile), Q2 (25th–50th percentile), Q3 (50th–75th percentile) and Q4 (75th–100th percentile). The univariate analysis was conducted to evaluate the independent effect of each predictor on the outcome. All potential covariates with p-value<=0.25 from univariate analysis were included in the multivariable and were dropped in a stepwise fashion based on statistical significance using purposeful selection. The variables having a p-value < 0.05 were retained in the final model. The data from logistic regression were presented as odds ratios with 95% confidence intervals.

## 3 Results

Out of 416 enrolled children, 400 received their first dose of RV vaccine along with collection of blood samples for RV IgA antibodies at a median age of 21 (IQR;16.2–25.9) weeks to serve as baseline for IgA. The second RV vaccine dose was administered to 391 RV1 vaccinated children at a median age of 26.7 (21.9–31.3) weeks.

### 3.1 Seroconversion status

**Fig. 2:**
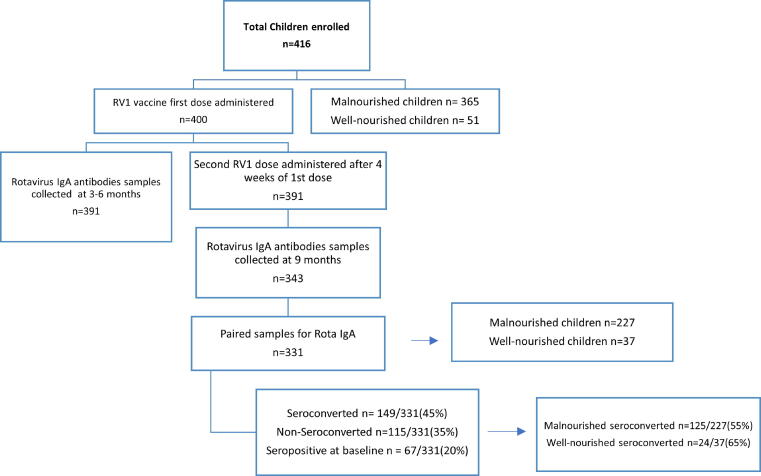
Participant’s seroconversion status at different stages of study. Abbreviations; RV1 = monovalent rotavirus vaccine, IgA = Immunoglobulin A.

Fig. 2 shows the seroconversion status after RV vaccination as evidenced by RV IgA levels measured at nine months. Children who provided the serum samples before and after vaccination were included in the analysis (n = 331). Of these, 149 (45%) seroconverted with a serum RV IgA > 20 AU/mL, 115 children (35%) did not seroconvert while 67 (20%) were already seropositive at baseline serum sampling. We did not see any boosting effect of seropositives at nine months. Seroconversion rate in malnourished children was 55% (n = 125/227) and in well-nourished children was 65% (n = 24/37, p-value = 0.260).

### 3.2 Baseline characteristics

Baseline characteristics of the children enrolled in our study at the time of birth is shown in Table 1. The demographic, socio-economic and nutritional status for seroconverted and non-seroconverted were comparable. The majority of births took place at a hospital (>75%). Most mothers (>85%) were illiterate, >97% of children were breast fed in both groups.

**Table 1:** Demographic data and seroconversion status of study population (n = 264) at the time of birth for enrolled infants.

	Seroconverted	Non-Sero converted	p-value
	N = 149	N = 115	
Age at registration (In days)	7.0 ± 6.4	7.4 ± 7.6	0.660
Gender			
Male	89 (59.7)	65 (56.5)	0.600
Female	60 (40.3)	50 (43.5)	
Place of birth			
Hospital	124 (83.8)	87 (75.7)	0.140
Home	23 (15.5)	28 (24.3)	
Enroute to hospital	1 (0.7)	0 (0.0)	
Child ever breastfed			
Yes	142 (97.3)	112 (99.1)	0.510
No	3 (2.1)	1 (0.9)	
Don’t Know	1 (0.7)	0 (0.0)	
Mother education			
No formal education/ Illiterate	125 (83.9)	97 (84.3)	0.920
Literate	24 (16.1)	18 (15.7)	
Age of mother	28.0 ± 6.7	29.8 ± 7.4	0.038
Family size	1.7 ± 0.5	1.7 ± 0.5	0.680
Poverty/wealth quintile index			
poorest	28 (18.8)	20 (17.4)	0.840
Poor	28 (18.8)	26 (22.6)	
Middle	30 (20.1)	20 (17.4)	
Rich	30 (20.1)	27 (23.5)	
Richest	33 (22.1)	22 (19.1)	
Household food insecurity index			
Food Secure	94 (63.1)	80 (69.6)	0.560
Mildly Food Insecure Access	5 (3.4)	5 (4.3)	
Moderately Food Insecure Access	23 (15.4)	12 (10.4)	
Severely Food Insecure Access	27 (18.1)	18 (15.7)	
Early initiation of breastfeeding			
Immediately after birth (first hour)	18 (12.6)	12 (10.6)	0.490
1–6 h after birth	75 (52.4)	62 (54.9)	
7–12 h after birth	27 (18.9)	14 (12.4)	
13–24 h after birth	13 (9.1)	16 (14.2)	
>24 h	10 (7.0)	9 (8.0)	
Wasting (Weight for Length)	34 (26.2)	23 (23.5)	0.640
WLZ, median (25th, 75th percentile)	−1.2 (-2.0, −0.5)	−1.1 (-2.0, −0.5)	0.750
Under-weight (Weight for Age)	60 (40.5)	53 (46.9)	0.300
WAZ, median (25th, 75th percentile)	−1.8 (-2.7, −1.1)	−1.9 (-2.5, −1.1)	0.470
Stunting (Length for Age)	47 (31.5)	41 (36.0)	0.450
LAZ median(25th, 75th percentile)	−1.6 (-2.1, −0.7)	−1.6 (-2.4, −0.9)	0.490

### 3.3 Growth anthropometrics and seroconversion

Non-seroconverted children were more likely to be stunted, wasted, and underweight at six months of age (Table 2). Growth parameters (mean WLZ, LAZ, and WAZ scores) were comparable between the two groups at 9 months.

**Table 2:** Association between anthropometric data and RV seroconversion status at baseline and nine months of age.

	At 6 months		P-values	At 9 months		P-values
	Sero converted	Non-Sero converted		Sero converted	Non-Sero converted	
Stunting (Length for Age)						
Severe Stunting (<-3)	35 (53.8)	30 (46.2)	0.056	30 (46.9)	34 (53.1)	0.290
Moderate Stunting(>-3 to < -2)	25 (43.9)	32 (56.1)		35 (59.3)	24 (40.7)	
Normal (>-2)	86 (62.3)	52 (37.7)		74 (57.4)	55 (42.6)	
LAZ(Mean ± SD)	−1.8 ± 1.3	−2.2 ± 1.3	0.010	−2.0 ± 1.3	−2.2 ± 1.3	0.240
n	146	114		139	113	
Wasting (Weight for Length)						
Severe Wasting (<-3)	24 (46.2)	28 (53.8)	0.120	17 (48.6)	18 (51.4)	0.710
Moderate Wasting(>-3 to < -2)	58 (54.2)	49 (45.8)		44 (56.4)	34 (43.6)	
Normal (>-2)	66 (62.9)	39 (37.1)		77 (55.8)	61 (44.2)	
WLZ(Mean ± SD)	−1.9 ± 1.4	−2.2 ± 1.2	0.034	−1.6 ± 1.3	−1.7 ± 1.2	0.350
n	148	116		138	113	
Under-weight (Weight for Age)						
Severe Under-weight (<-3)	54 (48.2)	58 (51.8)	0.043	41 (49)	42 (51)	0.190
Moderate Under-weight (>-3 and < -2	40 (56.3)	31 (43.7)		50 (63)	29 (37)	
Normal(>-2)	47 (67.1)	23 (32.9)		47 (53)	41 (47)	
WAZ(Mean ± SD)	−2.5 ± 1.5	−3.1 ± 1.4	<0.001	−2.3 ± 1.4	−2.6 ± 1.4	0.190
n	141	112		138	112	

### 3.4 EED biomarkers and change in seroconversion status

Next, we explored association of EE biomarkers and RV seroconversion assessed by IgA units. Table 3 shows the correlation of nine EE biomarkers with serum RV IgA units at baseline and at nine months of age. Serum RV IgA units were positively associated with pre-albumin (Spearman correlation r_s_ = 0.2114, *p*-value < 0.001) and AGP (r_s_ = 0.1054, *p*-value < 0.05) at the time of vaccine administration however, this association was lost at 9 months of age. IgA units measured at 9 months showed a weak negative but significant association with MPO (r_s_ = -0.1554, *p*-value < 0.05), a gut specific fecal inflammatory biomarker.

**Table 3:** Correlation between RV IgA units and inflammatory biomarkers at baseline and 9 months of age.

	Correlation coefficient	
Biomarkers	With baseline IgA units	With IgA units at 9 months
GLP	0.0088	0.008
NEO	−0.0442	−0.0204
MPO	0.0085	−0.1554*
AGP	0.1054*	−0.0124
Ferritin	−0.0062	−0.0357
CRP	−0.0531	0.0107
Leptin	−0.0714	−0.0609
IGF	−0.0702	−0.0398
Pre-Albumin	0.2114**	−0.089
***p-value < 0.0001; ** p-value < 0.001; *p value < 0.05		

Abbreviations; myeloperoxidase (MPO), Glucagon like peptide (GLP), C-reactive protein (CRP), Insulin like growth factor (IGF-1), α1-acid glycoprotein (IGF) and neopterin (NEO).

Next, we compared the levels of biomarkers before RV vaccination administration and at nine months between seroconverted and non-seroconverted children (Table 4). The seroconverted children reported to have higher AGP levels at the time of vaccination (p = 0.077) which was also observed in the case of IGF-1 that was significantly higher in seroconverters (p-value = 0.024). However, at nine months only MPO showed a significant difference in seroconverted [3050.0 ng/ml (1250, 7587)] and non-seroconverted [4623.3 ng/ml (2189, 11650)] children respectively (p = 0.017). As we explored change in the level of biomarkers from the time of vaccine administration to 9 months of age, no significant trend was observed (Table 5). The multivariable analysis (data in supplementary tables) showed that serum IGF-1 and AGP levels at the time of vaccine administration were positively associated with the probability of RV seroconversion. The highest quartile of AGP and IGF-1 was associated with an increased likelihood of seroconversion compared to the lowest quartile (AGP: OR = 2.78, 95% CI: 1.32–5.84 and IGF: OR = 3.39, 95% CI: 1.58–7.30) (Supplementary Table S1). The biomarkers at nine months were not significantly associated with seroconversion (Supplementary table S2).

**Table 4:** Comparison of gut-specific and systemic inflammatory biomarkers between seroconverters and non-seroconverters.

Biomarkers	Seroconverted (n = 149)	Non-Seroconverted (n = 115)	p-value^¥^
At 6 months			
GLP (pg/ml)	989.2 (720.1–1548.2)	1016.7 (703.9–1453.7)	0.850
NEO (nmol/L)	2100.0 (1037.5–3502.6)	1834.5 (990.0–2650.0)	0.170
MPO (ng/ml)	8725.5 (2900.0–19535.3)	8800.0 (3050.0–18000.0)	0.960
AGP(mg/l)	89.0 (68.4–123.0)	82.6 (58.0–108.2)	0.077
Ferritin(ng/ml)	79.0 (33.0–172.0)	85.0 (32.0–178.6)	0.950
CRP (mg/l)	0.1 (0.1–0.4)	0.1 (0.1–0.3)	0.590
Leptin (pg/ml)	174.0 (87.5–293.9)	144.1 (76.1–262.6)	0.140
IGF-1(ng/ml)	26.3 (16.5–43.5)	22.5 (13.6–36.3)	0.024
Pre-Albumin (mg/l)	13.8 (11.7–16.0)	13.6 (12.0–16.1)	0.970
At 9 months			
GLP (pg/ml)	1211.4 (835.4–1834.9)	1260.9 (774.3–1705.9)	0.63
NEO (nmol/L)	1975.0 (927.4–2625.0)	1800.0 (875.0–2850.0)	0.88
MPO (ng/ml)	3050.0 (1250.0–7587.0)	4623.3 (2189.0–11650.0)	0.017
AGP (mg/l)	104.0 (77.0–130.4)	97.0 (76.0–139.0)	0.93
Ferritin(ng/ml)	15.0 (6.2–36.7)	14.4 (7.0–37.0)	0.55
CRP (mg/l)	0.2 (0.1–0.4)	0.2 (0.1–0.3)	0.96
Leptin (pg/ml)	176.1 (109.5–294.3)	184.8 (109.3–326.7)	0.81
IGF-1 (ng/ml)	20.6 (14.6–33.6)	20.2 (11.9–33.7)	0.58
Pre-Albumin (mg/l)	14.0 (12.0–16.3)	14.3 (12.2–17.3)	0.25
*Concentrations shown as medians with interquartile ranges			
^¥^Mann–Whitney *U* test.

**Table 5:** Change in gut-specific and systemic inflammatory biomarkers concentrations between the seroconverters and non-seroconverters.

Biomarkers	Seroconverted (n = 149)	Non-Seroconverted (n = 115)	p-value^¥^
Δ GLP (pg/ml)	146.3 (-211.2–551.7)	124.2 (-328.8–538.8)	0.70
Δ NEO (nmol/L)	−79.9 (-1237.5–932.5)	−75.0 (-925.0–1006.8)	0.37
Δ MPO (ng/ml)	−2548.8 (-12959.8–1401.8)	−1804.3 (-9782.0–3733.5)	0.21
Δ AGP (mg/l)	7.7 (-21.5–44.7)	17.0 (-17.0–52.0)	0.14
Δ Ferritin(ng/ml)	−54.0 (-133.0--15.0)	−54.0 (-131.0--9.0)	0.61
Δ CRP (mg/l)	0.004 (-0.2–0.3)	0.039 (-0.2–0.2)	0.49
Δ Leptin (pg/ml)	13.3 (-88.9–101.9)	27.4 (-70.5–93.5)	0.41
Δ IGF (ng/ml)	−5.9 (–22.2–8.3)	−3.0 (-16.1–11.6)	0.19
Δ Pre-Albumin (mg/l)	0.2 (-1.7–2.5)	1.0 (-2.2–3.0)	0.60

Abbreviations; myeloperoxidase (MPO), calprotectin (CAL), Glucagon like peptide (GLP), C-reactive protein(CRP), Insulin like growth factor(IGF-1), α1-acid glycoprotein(IGF) and neopterin(NEO). Concentration difference shown as median (IQR) between baseline readings and 9 months.

### 3.5 Fecal pathogens and RV seroconversion

Lastly, we explored association of presence of enteropathogen in non-diarrheal stool samples collected at the time of administration of the vaccine (Table 6). Sapovirus detection was significantly associated with non-seroconversion in our study. On the other hand, RV was detected more commonly in fecal samples of children who later seroconverted (p = 0.055), however no difference was seen in the prevalence of Giardia between the two groups. Detailed analysis of all pathogens at enrollment and nine months is available in Supplementary table S3.

**Table 6:** Pathogen detection in non-diarrheal stool samples collected at the time of RV first dose administration. The data is segregated on the bases of seroconversion status.

Fecal pathogen	At the time of enrollment		
	Sero converted	Non-Sero converted	p-value
	N = 143	N = 104	
Astrovirus	8 (5.6%)	11 (10.6%)	0.15
Sapovirus	12 (8.4%)	18 (17.3%)	0.03
Shigella	21 (14.7%)	12 (11.5%)	0.47
Adenovirus_40_41	14 (9.8%)	11 (10.6%)	0.84
Rotavirus*	28 (19.6%)	11 (10.6%)	0.06
Cryptosporidium	22 (15.4%)	15 (14.4%)	0.83
ETEC	37 (25.9%)	31 (29.8%)	0.49
Norovirus_GII	40 (28.0%)	22 (21.2%)	0.22
EPEC	48 (33.6%)	36 (34.6%)	0.86
Giardia	71 (49.7%)	49 (47.1%)	0.69
Campylobacter	89 (62.2%)	73 (70.2%)	0.19
Enteroaggregative Escherichia coli (EAEC)	99 (69.2%)	70 (67.3%)	0.750

Out of 27 protozoal, bacterial and viral targets tested, this table includes pathogens that were detected in at least 5% the stool samples.

* RV association with seroconversion was primarily from detecting rotavirus in the stool in the aftermath of the first vaccine dose.

## 4 Discussion

This study explores the relationship between growth parameters, markers of gut-specific and systemic inflammation, enteropathogen burden and RV seroconversion among children living in EED endemic settings. In a rural Pakistani population, we report 45% seroconversion similar to the range of 39–59% reported in other low- and middle-income countries (LMICs), including South Africa, India, and Malawi [4], [5], [6]. The seroconversion rate was alike for our study participants recruited as undernourished cases as well as controls. In various studies, the number of diarrhea episodes attributed to RV infection have been used to measure vaccine efficacy where malnourished and well-nourished children have shown similar efficacy[4], [5], [6]. Bangladeshi study found a positive correlation between markers of EE with vaccine failure but found no demonstrable link between nutritional status alone and seroconversion [8]. These findings were replicated in a study of infants in Brazil, Mexico, and Venezuela where no noticeable differences were seen in the reduction of diarrhea episodes after oral RV vaccine administration based on nutritional status [36].

Regarding association with growth, children who failed to seroconvert had a higher prevalence of stunting at 6 months of age. In addition, they had lower WLZ scores and WHZ scores suggestive of being wasted and underweight. However, these children seemed to display the phenomenon of “catch-up” growth as their growth parameters did not show any significant differences between the groups at nine months of age (Table 2). In a study of 219 Zimbabwean children, oral RV seroconversion was positively associated with a higher LAZ score around the time of vaccination suggesting implications of intrauterine growth restriction as poor vaccine response [37]. In Peru children vaccinated with RV vaccine had higher HAZ scores [38], potentially a consequence of improved nutritional status due to decreased RV diarrheal episodes. Systemic inflammation due to diarrhea can cause malabsorption that leads to suppression of growth and further increases the risk of oral vaccine underperformance. In this study, we explored the relationship between seroconversion and inflammatory biomarkers, yet we did not consider diarrheal episodes in our population.

Pre-albumin levels at the time of vaccine administration positively correlated with IgA baseline levels (r = 0.2114) and AGP (r = 0.1054). Pre-albumin, synthesized by the liver and gastrointestinal mucosais a marker of protein malnutrition. Although we did not find difference in overall vaccine response in nourished versus malnourished groups, the positive correlation between pre-albumin and vaccine response suggests that protein malnutrition may play a role in the vaccine response. In a cohort of 246 children from Bangladesh, fecal loss of protein biomarkers such as MPO, neopterin, and alpha-1-antitrypsin hints at subclinical mucosal inflammation in EE affecting barrier integrity, potentially leading to protein wasting in the stool [26]. In turn, protein malnutrition leads to decreased growth in childhood and potentially decreased immune response to vaccines.

In our analyses, serum IGF at the time of enrollment (3 to 6 months) was significantly higher in seroconverters supporting better response to RV vaccine in children with healthier growth trajectory, however, this trend was not seen at a later age suggesting growth catch-up via other mechanisms. Arndt et al noted a degree of positive correlation between lower levels of serum IGF with both growth faltering as well as decreased vaccine response, in concordance with our study [39].

At nine months, we found a negative association of fecal MPO with serum RV IgA but not with neopterin. Fecal MPO is a marker of neutrophilic inflammation in various inflammatory bowel conditions and is linked to chronic inflammation in EED that may be a driver hindering with vaccine immunogenicity [27], [25]. Additionally, as a gut-specific inflammatory marker, MPO has been negatively correlated with short-term growth faltering in infants [39]. In Nicaragua infants, three times higher median concentrations of MPO were reported in children who failed to seroconvert after administration of RV vaccine [40]. Neopterin, another biomarker of EED is secreted by macrophages in inflammation in response to interferon-y [28]. Previous literature such as the MAL-ED study and PROVIDE studies have shown its association with decreased growth and RV seroconversion yet we did not see this trend in our study [8].

Lastly, while exploring the role of fecal enteropathogen on seroconversion, although there was a high prevalence of pathogen burden, yet no difference was seen in seroconverters and non-seroconverters. The prevalence of bacteria, protozoa and viruses in non-diarrheal stools collected at the time of vaccine administration were similar in both the groups. This was even true while comparing the burden between undernourished cases and controls (data under-review) suggesting equitable pathogen exposure to healthy growers as well as poor growers. Thus, through our extensive analysis, we found no link between enteropathogen burden and seroconversion as we had hypothesized. In line to our study, a Zimbabwean study, a high prevalence of enteropathogen infections at the time of oral RV vaccine administration was seen. However, the authors found no definable relationship between RV vaccine immunogenicity and enteropathogen burden and did not see an improvement in vaccine response after improved sanitation and hygiene practices [41]. On the contrary, in a Bangladeshi cohort, the authors found association of enterovirus quantity with diminished RV IgA, failure to seroconvert, as well as increased RV associated diarrheal events [9].

Our study is an effort to examine RV seroconversion in the context of EED through the analysis of multiple factors including monthly growth data that is previously related to this asymptomatic condition. Additionally, this study led to administration of RV vaccine doses to children living in a setting where RV vaccination was not yet part of the national immunization program. However, our study is limited as we did not explore reduction in RV associated diarrhea as an outcome while seroconversion may not be a reliable measure of protection and vaccine response. In addition, this was a single rural site study, and our findings might not be generalizable to other settings. Lastly, our study describes a seroconversion rate of 45% while measuring IgA units at 9 months of age that was up to 3 months after administration of the second dose increasing a risk of adding potential RV infections that occurred between these time points as an added reason for higher IgA units. Lastly, gut specific biomarkers were collected as a part of the parent SEEM study for EED, with no a priori hypotheses surrounding RV vaccine immunogenicity and specific biomarkers. That was he limitation, so we have tried to find the association of RV seroconversion with the biomarkers that were available with us.

In conclusion, we showed a complex relationship of EED with oral vaccine failure. The response of RV vaccines in developing countries is better than LMIC, as shown by reduced RV-related hospitalizations and deaths in children under five [42], [43]. Vaccine failure in LMIC s multifactorial, and EED is one of the factors if not the sole reason of vaccine failure. A multipronged approach will be needed to better characterize pathological mechanism of EED using novel biomarkers, nutritional, and educational interventions.

## 5 Ethics approval and consent to participate.

Aga Khan University Hospital’s ethical review committee approved the study in 2015 with ERC number 3836-Ped-ERC-15. Written informed consent was obtained from parents/guardians of enrolled children.

## Funding

The study was funded by the Bill and Melinda Gates foundation (AA: OPP1138727, SRM: OPP1144149). AA and SRM also received funding from Fogarty International Center (D43TW007585). Additionally, SRM received funding from Pendleton Laboratory Endowment funds. The funding sources had no role in the design of the study and collection, analysis, and interpretation of data and in writing this manuscript.

## Declaration of Competing Interest

The authors declare that they have no known competing financial interests or personal relationships that could have appeared to influence the work reported in this paper.
