# Supplementary material for: Association of Anti-Rotavirus IgA Seroconversion with Growth, Environmental Enteric Dysfunction and Enteropathogens in Rural Pakistani Infants
Source: Vaccine. 2022 May 31;40(25):3444–51. doi: 10.1016/j.vaccine.2022.04.032 (PMC9168439; doi:10.1016/j.vaccine.2022.04.032)
Supplement: Supplementary data 1 [file mmc1.docx]

**Supplementary Table S-1. Logistics regression modeling results: Bivariate and multivariable determinants of seroconversion status at enrollment**

| \| **Characteristics** \| **Unadjusted OR (95% CI)** \| **p-value** \| **Adjusted OR (95% CI)** \| **p-value** \| \| --- \| --- \| --- \| --- \| --- \| \| **Age at registration (In months)** \| 0.784 (0.269,2.283) \| 0.656 \|  \|  \| \| **Gender** \|  \|  \|  \|  \| \| Male \| 0.992 (0.958,1.027) \| 0.656 \|  \|  \| \| Female \| Ref. \|  \|  \|  \| \| **Place of birth** \|  \|  \|  \|  \| \| Hospital \| 1.663 (0.903,3.061) \| 0.102 \|  \|  \| \| Home/Enroute to hospital \| Ref. \|  \|  \|  \| \| **Child ever breastfed** \|  \|  \|  \|  \| \| Yes \| 0.317 (0.035,2.876) \| 0.307 \|  \|  \| \| No/Don't Know \| Ref. \|  \|  \|  \| \| **Mother education** \|  \|  \|  \|  \| \| No formal education/ Illiterate \| 0.966 (0.496,1.882) \| 0.92 \|  \|  \| \| Literate \| Ref. \|  \|  \|  \| \| **Age of mother** \| 0.964 (0.931,0.998) \| 0.040 \|  \|  \| \| **Family size** \| 0.891 (0.521,1.525) \| 0.674 \|  \|  \| \| **Poverty/wealth quintile index** \|  \|  \|  \|  \| \| Poorest \| 0.947 (0.552,1.625) \| 0.843 \|  \|  \| \| Middle \| 1.167 (0.592,2.298) \| 0.656 \|  \|  \| \| Two Richest \| Ref. \|  \|  \|  \| \| **Household food insecurity index** \|  \|  \|  \|  \| \| Food Secure/Mildly Food Insecure Access \| Ref. \|  \|  \|  \| \| Moderately to Severely Food Insecure Access \| 1.431 (0.836,2.449) \| 0.191 \|  \|  \| \| **Early initiation of breastfeeding** \|  \|  \|  \|  \| \| Yes \| 1.212 (0.558,2.634) \| 0.627 \|  \|  \| \| No \| Ref. \|  \|  \|  \| \| **Wasting (Weight for Height)** \|  \|  \|  \|  \| \| Wasted \| 1.155 (0.628,2.124) \| 0.643 \|  \|  \| \| Normal \| Ref. \|  \|  \|  \| \| **Under-weight (Weight for Age)** \|  \|  \|  \|  \| \| Under-weight \| 0.772 (0.471,1.265) \| 0.304 \|  \|  \| \| Normal \| Ref. \|  \|  \|  \| \| **Stunting (Height for Age)** \|  \|  \|  \|  \| \| Stunted \| 0.82 (0.49,1.374) \| 0.452 \|  \|  \| \| Normal \| Ref. \|  \|  \|  \| \| **Biomarkers from 6 months** \|  \|  \|  \|  \| \| **GLP (pg/ml)** \|  \|  \|  \|  \| \| Q1 \| Ref. \|  \|  \|  \| \| Q2 \| 1.235 (0.621,2.456) \| 0.548 \|  \|  \| \| Q3 \| 0.941 (0.475,1.865) \| 0.861 \|  \|  \| \| Q4 \| 1.167 (0.576,2.363) \| 0.669 \|  \|  \| \| **NEO (nmol/L)** \|  \|  \|  \|  \| \| Q1 \| Ref. \|  \|  \|  \| \| Q2 \| 0.767 (0.376,1.563) \| 0.465 \|  \|  \| \| Q3 \| 1.064 (0.53,2.135) \| 0.862 \|  \|  \| \| Q4 \| 1.448 (0.719,2.915) \| 0.3 \|  \|  \| \| **MPO (ng/ml)** \|  \|  \|  \|  \| \| Q1 \| Ref. \|  \|  \|  \| \| Q2 \| 0.993 (0.488,2.018) \| 0.984 \|  \|  \| \| Q3 \| 0.872 (0.434,1.754) \| 0.702 \|  \|  \| \| Q4 \| 1.055 (0.518,2.149) \| 0.882 \|  \|  \| \| **AGP (mg/l)** \|  \|  \|  \|  \| \| Q1 \| Ref. \|  \| Ref. \|  \| \| Q2 \| 1.785 (0.913,3.489) \| 0.09 \| 1.989 (0.999,3.959) \| 0.050 \| \| Q3 \| 1.755 (0.878,3.506) \| 0.111 \| 1.943 (0.952,3.965) \| 0.068 \| \| Q4 \| 2.12 (1.059,4.243) \| 0.034 \| 2.781 (1.324,5.841) \| 0.007 \| \| **Ferritin(ng/ml)** \|  \|  \|  \|  \| \| Q1 \| Ref. \|  \|  \|  \| \| Q2 \| 1.465 (0.73,2.937) \| 0.283 \|  \|  \| \| Q3 \| 0.954 (0.484,1.884) \| 0.893 \|  \|  \| \| Q4 \| 1.039 (0.53,2.036) \| 0.912 \|  \|  \| \| **CRP (mg/l)** \|  \|  \|  \|  \| \| Q1 \| Ref. \|  \|  \|  \| \| Q2 \| 0.695 (0.351,1.378) \| 0.298 \|  \|  \| \| Q3 \| 0.802 (0.407,1.579) \| 0.522 \|  \|  \| \| Q4 \| 1.086 (0.532,2.217) \| 0.821 \|  \|  \| \| **Leptin (pg/ml)** \|  \|  \|  \|  \| \| Q1 \| Ref. \|  \|  \|  \| \| Q2 \| 1.097 (0.543,2.213) \| 0.797 \|  \|  \| \| Q3 \| 1.434 (0.712,2.888) \| 0.313 \|  \|  \| \| Q4 \| 1.526 (0.756,3.081) \| 0.238 \|  \|  \| \| **IGF (ng/ml)** \|  \|  \|  \|  \| \| Q1 \| Ref. \|  \| Ref. \|  \| \| Q2 \| 1.57 (0.772,3.194) \| 0.213 \| 1.814 (0.874,3.765) \| 0.110 \| \| Q3 \| 1.462 (0.726,2.941) \| 0.287 \| 1.811 (0.872,3.76) \| 0.111 \| \| Q4 \| 2.629 (1.275,5.423) \| 0.009 \| 3.398 (1.581,7.305) \| 0.002 \| \| **Pre-Albumin (mg/l)** \|  \|  \|  \|  \| \| Q1 \| Ref. \|  \|  \|  \| \| Q2 \| 0.677 (0.343,1.333) \| 0.259 \|  \|  \| \| Q3 \| 1.1 (0.534,2.264) \| 0.797 \|  \|  \| \| Q4 \| 0.996 (0.477,2.083) \| 0.992 \|  \|  \|   **Supplementary Table S-2. Logistics regression modeling results: Bivariate and multivariable determinants of seroconversion status at 9 months** | | | | |
| --- | --- | --- | --- | --- | --- | --- | --- | --- | --- | --- | --- | --- | --- | --- | --- | --- | --- | --- | --- | --- | --- | --- | --- | --- | --- | --- | --- | --- | --- | --- | --- | --- | --- | --- | --- | --- | --- | --- | --- | --- | --- | --- | --- | --- | --- | --- | --- | --- | --- | --- | --- | --- | --- | --- | --- | --- | --- | --- | --- | --- | --- | --- | --- | --- | --- | --- | --- | --- | --- | --- | --- | --- | --- | --- | --- | --- | --- | --- | --- | --- | --- | --- | --- | --- | --- | --- | --- | --- | --- | --- | --- | --- | --- | --- | --- | --- | --- | --- | --- | --- | --- | --- | --- | --- | --- | --- | --- | --- | --- | --- | --- | --- | --- | --- | --- | --- | --- | --- | --- | --- | --- | --- | --- | --- | --- | --- | --- | --- | --- | --- | --- | --- | --- | --- | --- | --- | --- | --- | --- | --- | --- | --- | --- | --- | --- | --- | --- | --- | --- | --- | --- | --- | --- | --- | --- | --- | --- | --- | --- | --- | --- | --- | --- | --- | --- | --- | --- | --- | --- | --- | --- | --- | --- | --- | --- | --- | --- | --- | --- | --- | --- | --- | --- | --- | --- | --- | --- | --- | --- | --- | --- | --- | --- | --- | --- | --- | --- | --- | --- | --- | --- | --- | --- | --- | --- | --- | --- | --- | --- | --- | --- | --- | --- | --- | --- | --- | --- | --- | --- | --- | --- | --- | --- | --- | --- | --- | --- | --- | --- | --- | --- | --- | --- | --- | --- | --- | --- | --- | --- | --- | --- | --- | --- | --- | --- | --- | --- | --- | --- | --- | --- | --- | --- | --- | --- | --- | --- | --- | --- | --- | --- | --- | --- | --- | --- | --- | --- | --- | --- | --- | --- | --- | --- | --- | --- | --- | --- | --- | --- | --- | --- | --- | --- | --- | --- | --- | --- | --- | --- | --- | --- | --- | --- | --- | --- | --- | --- | --- | --- | --- | --- | --- | --- | --- | --- | --- | --- | --- | --- | --- | --- | --- | --- | --- | --- | --- | --- | --- | --- | --- | --- | --- | --- | --- | --- | --- | --- | --- | --- | --- | --- | --- | --- | --- | --- | --- | --- | --- | --- | --- | --- | --- | --- | --- | --- | --- | --- | --- | --- | --- | --- | --- | --- | --- | --- | --- | --- | --- | --- | --- | --- | --- | --- | --- | --- | --- | --- | --- | --- | --- | --- | --- | --- | --- | --- | --- | --- | --- | --- | --- | --- | --- | --- | --- | --- | --- | --- | --- | --- | --- | --- | --- | --- | --- | --- | --- | --- | --- | --- | --- | --- | --- | --- | --- | --- | --- | --- | --- | --- |
| **Baseline Characteristics** | **Unadjusted OR (95 CI%)** | **p-value** | **Adjusted OR (95 CI%)** | **p-value** |
| **Age at registration (In months)** | 0.784 (0.269,2.283) | 0.656 |  |  |
| **Gender** |  |  |  |  |
| Male | 0.992 (0.958,1.027) | 0.656 |  |  |
| Female | Ref. |  |  |  |
| **Place of birth** |  |  |  |  |
| Hospital | 1.663 (0.903,3.061) | 0.102 | 0.45 (0.229,0.883) | 0.020 |
| Home/Enroute to hospital | Ref. |  | Ref. |  |
| **Child ever breastfed** |  |  |  |  |
| Yes | 0.317 (0.035,2.876) | 0.307 |  |  |
| No/Don't Know | Ref. |  |  |  |
| **Mother education** |  |  |  |  |
| No formal education/ Illiterate | 0.966 (0.496,1.882) | 0.92 |  |  |
| Literate | Ref. |  |  |  |
| **Age of mother** | 0.964 (0.931,0.998) | 0.040 |  |  |
| **Family size** | 0.891 (0.521,1.525) | 0.674 |  |  |
| **Poverty/wealth quintile index** |  |  |  |  |
| Poorest | 0.947 (0.552,1.625) | 0.843 |  |  |
| Middle | 1.167 (0.592,2.298) | 0.656 |  |  |
| Two Richest | Ref. |  |  |  |
| **Household food insecurity index** |  |  |  |  |
| Food Secure/Mildly Food Insecure Access | Ref. |  | Ref. |  |
| Moderately to Severely Food Insecure Access | 1.431 (0.836,2.449) | 0.191 | 1.88 (1.046,3.378) | 0.035 |
| **Early initiation of breastfeeding** |  |  |  |  |
| Yes | 1.212 (0.558,2.634) | 0.627 |  |  |
| No | Ref. |  |  |  |
| **Wasting (Weight for Height)** |  |  |  |  |
| Wasted | 1.155 (0.628,2.124) | 0.643 |  |  |
| Nomal | Ref. |  |  |  |
| **Under-weight (Weight for Age)** |  |  |  |  |
| Under-weight | 0.772 (0.471,1.265) | 0.304 |  |  |
| Nomal | Ref. |  |  |  |
| **Stunting (Height for Age)** |  |  |  |  |
| Stunted | 0.82 (0.49,1.374) | 0.452 |  |  |
| Nomal | Ref. |  |  |  |
| **Δ Concentration of Biomarkers from 6 to 9 months** |  |  |  |  |
| **GLP (pg/ml)** |  |  |  |  |
| Q1 | Ref. |  |  |  |
| Q2 | 1.491 (0.719,3.094) | 0.283 |  |  |
| Q3 | 1.406 (0.696,2.841) | 0.342 |  |  |
| Q4 | 1.238 (0.602,2.542) | 0.562 |  |  |
| **NEO (nmol/L)** |  |  |  |  |
| Q1 | Ref. |  |  |  |
| Q2 | 0.731 (0.365,1.465) | 0.377 |  |  |
| Q3 | 0.881 (0.438,1.771) | 0.722 |  |  |
| Q4 | 0.844 (0.424,1.683) | 0.631 |  |  |
| **MPO (ng/ml)** |  |  |  |  |
| Q1 | Ref. |  |  |  |
| Q2 | 0.929 (0.464,1.859) | 0.834 |  |  |
| Q3 | 0.914 (0.453,1.843) | 0.801 |  |  |
| Q4 | 0.667 (0.334,1.332) | 0.251 |  |  |
| **AGP (mg/l)** |  |  |  |  |
| Q1 | Ref. |  |  |  |
| Q2 | 1.045 (0.516,2.116) | 0.904 |  |  |
| Q3 | 0.695 (0.341,1.415) | 0.315 |  |  |
| Q4 | 0.67 (0.333,1.346) | 0.261 |  |  |
| **Ferritin(ng/ml)** |  |  |  |  |
| Q1 | Ref. |  |  |  |
| Q2 | 0.9 (0.453,1.79) | 0.764 |  |  |
| Q3 | 1.214 (0.6,2.457) | 0.59 |  |  |
| Q4 | 0.711 (0.354,1.424) | 0.335 |  |  |
| **CRP (mg/l)** |  |  |  |  |
| Q1 | Ref. |  | Ref. |  |
| Q2 | 0.696 (0.343,1.412) | 0.316 | 0.639 (0.304,1.343) | 0.237 |
| Q3 | 0.497 (0.246,1.004) | 0.051 | 0.446 (0.21,0.945) | 0.035 |
| Q4 | 0.885 (0.436,1.797) | 0.735 | 0.875 (0.408,1.879) | 0.733 |
| **Leptin (pg/ml)** |  |  |  |  |
| Q1 | Ref. |  |  |  |
| Q2 | 0.917 (0.439,1.916) | 0.817 |  |  |
| Q3 | 0.731 (0.36,1.487) | 0.387 |  |  |
| Q4 | 0.972 (0.467,2.023) | 0.94 |  |  |
| **IGF (ng/ml)** |  |  |  |  |
| Q1 | Ref. |  | Ref. |  |
| Q2 | 0.545 (0.269,1.106) | 0.093 | 0.47 (0.223,0.993) | 0.048 |
| Q3 | 0.602 (0.296,1.227) | 0.163 | 0.556 (0.265,1.164) | 0.120 |
| Q4 | 0.559 (0.279,1.119) | 0.101 | 0.51 (0.245,1.064) | 0.073 |
| **Pre-Albumin (mg/l)** |  |  |  |  |
| Q1 | Ref. |  |  |  |
| Q2 | 1.618 (0.772,3.39) | 0.203 |  |  |
| Q3 | 1.172 (0.569,2.418) | 0.667 |  |  |
| Q4 | 1.065 (0.516,2.197) | 0.866 |  |  |

**Supplementary table S-3: Comparison of Fecal pathogens TAC data with seroconversion status at enrollment and nine months**

|  |  |  |  |  |  |  |  |
| --- | --- | --- | --- | --- | --- | --- | --- |
| **Fecal pathogen TAC data** | **TAC at 6 months** | | |  | **TAC at 9 months** | | |
|  | **Sero converted** | **Non-Sero converted** | **p-value** |  | **Sero converted** | **Non-Sero converted** | **p-value** |
|  | **N=143** | **N=104** |  |  | **N=143** | **N=104** |  |
| **Aeromonas** |  |  | 0.180 |  |  |  | 0.890 |
| Yes | 1 ( 0.7%) | 3 ( 2.9%) |  |  | 6 ( 4.2%) | 4 ( 3.8%) |  |
| No | 142 (99.3%) | 101 (97.1%) |  |  | 137 (95.8%) | 100 (96.2%) |  |
| **Cryptosporidium** |  |  | 0.830 |  |  |  | 0.360 |
| Yes | 22 (15.4%) | 15 (14.4%) |  |  | 30 (21.0%) | 27 (26.0%) |  |
| No | 121 (84.6%) | 89 (85.6%) |  |  | 113 (79.0%) | 77 (74.0%) |  |
| **Campylobacter** |  |  | 0.190 |  |  |  | 0.840 |
| Yes | 89 (62.2%) | 73 (70.2%) |  |  | 114 (79.7%) | 84 (80.8%) |  |
| No | 54 (37.8%) | 31 (29.8%) |  |  | 29 (20.3%) | 20 (19.2%) |  |
| **EAEC** |  |  | 0.750 |  |  |  | 0.390 |
| Yes | 99 (69.2%) | 70 (67.3%) |  |  | 110 (76.9%) | 75 (72.1%) |  |
| No | 44 (30.8%) | 34 (32.7%) |  |  | 33 (23.1%) | 29 (27.9%) |  |
| **EPEC** |  |  | 0.860 |  |  |  | 0.960 |
| Yes | 48 (33.6%) | 36 (34.6%) |  |  | 71 (49.7%) | 52 (50.0%) |  |
| No | 95 (66.4%) | 68 (65.4%) |  |  | 72 (50.3%) | 52 (50.0%) |  |
| **ETEC** |  |  | 0.490 |  |  |  | 0.460 |
| Yes | 37 (25.9%) | 31 (29.8%) |  |  | 49 (34.3%) | 31 (29.8%) |  |
| No | 106 (74.1%) | 73 (70.2%) |  |  | 94 (65.7%) | 73 (70.2%) |  |
| **H_pylori** |  |  | 0.390 |  |  |  | 0.820 |
| Yes | 1 ( 0.7%) | 0 ( 0.0%) |  |  | 1 ( 0.7%) | 1 ( 1.0%) |  |
| No | 142 (99.3%) | 104 (100.0%) |  |  | 142 (99.3%) | 103 (99.0%) |  |
| **M_tuberculosis** |  |  | 0.480 |  |  |  | 0.750 |
| Yes | 3 ( 2.1%) | 1 ( 1.0%) |  |  | 2 ( 1.4%) | 2 ( 1.9%) |  |
| No | 140 (97.9%) | 103 (99.0%) |  |  | 141 (98.6%) | 102 (98.1%) |  |
| **Salmonella** |  |  | 0.390 |  |  |  | 0.240 |
| Yes | 1 ( 0.7%) | 0 ( 0.0%) |  |  | 0 ( 0.0%) | 1 ( 1.0%) |  |
| No | 142 (99.3%) | 104 (100.0%) |  |  | 143 (100.0%) | 103 (99.0%) |  |
| **Shigella** |  |  | 0.470 |  |  |  | 0.130 |
| Yes | 21 (14.7%) | 12 (11.5%) |  |  | 22 (15.4%) | 24 (23.1%) |  |
| No | 122 (85.3%) | 92 (88.5%) |  |  | 121 (84.6%) | 80 (76.9%) |  |
| **Plesiomonas** |  |  |  |  |  |  | 0.760 |
| Yes | 0 ( 0.0%) | 0 ( 0.0%) | - |  | 2 ( 1.4%) | 1 ( 1.0%) |  |
| No | 143 (100.0%) | 104 (100.0%) |  |  | 141 (98.6%) | 103 (99.0%) |  |
| **Adenovirus_40_41** |  |  | 0.840 |  |  |  | 0.330 |
| Yes | 14 ( 9.8%) | 11 (10.6%) |  |  | 29 (20.3%) | 16 (15.4%) |  |
| No | 129 (90.2%) | 93 (89.4%) |  |  | 114 (79.7%) | 88 (84.6%) |  |
| **Astrovirus** |  |  | 0.150 |  |  |  | 0.740 |
| Yes | 8 ( 5.6%) | 11 (10.6%) |  |  | 12 ( 8.4%) | 10 ( 9.6%) |  |
| No | 135 (94.4%) | 93 (89.4%) |  |  | 131 (91.6%) | 94 (90.4%) |  |
| **Norovirus_GI** |  |  | 0.570 |  |  |  | 0.017 |
| Yes | 6 ( 4.2%) | 6 ( 5.8%) |  |  | 18 (12.6%) | 4 ( 3.8%) |  |
| No | 137 (95.8%) | 98 (94.2%) |  |  | 125 (87.4%) | 100 (96.2%) |  |
| **Norovirus_GII** |  |  | 0.220 |  |  |  | 0.220 |
| Yes | 40 (28.0%) | 22 (21.2%) |  |  | 49 (34.3%) | 28 (26.9%) |  |
| No | 103 (72.0%) | 82 (78.8%) |  |  | 94 (65.7%) | 76 (73.1%) |  |
| **Rotavirus** |  |  | 0.055 |  |  |  | 0.022 |
| Yes | 28 (19.6%) | 11 (10.6%) |  |  | 7 ( 4.9%) | 0 ( 0.0%) |  |
| No | 115 (80.4%) | 93 (89.4%) |  |  | 136 (95.1%) | 104 (100.0%) |  |
| **Sapovirus** |  |  | 0.034 |  |  |  | 0.760 |
| Yes | 12 ( 8.4%) | 18 (17.3%) |  |  | 34 (23.8%) | 23 (22.1%) |  |
| No | 131 (91.6%) | 86 (82.7%) |  |  | 109 (76.2%) | 81 (77.9%) |  |
| **Ancylostoma** |  |  | - |  |  |  | - |
| No | 143 (100.0%) | 104 (100.0%) |  |  | 143 (100.0%) | 104 (100.0%) |  |
| **Ascaris** |  |  | - |  |  |  | - |
| No | 143 (100.0%) | 104 (100.0%) |  |  | 143 (100.0%) | 104 (100.0%) |  |
| **Strongyloides** |  |  | - |  |  |  | - |
| No | 143 (100.0%) | 104 (100.0%) |  |  | 143 (100.0%) | 104 (100.0%) |  |
| **Trichuris** |  |  | - |  |  |  | - |
| No | 143 (100.0%) | 104 (100.0%) |  |  | 143 (100.0%) | 104 (100.0%) |  |
| **Necator** |  |  | - |  |  |  | - |
| No | 143 (100.0%) | 104 (100.0%) |  |  | 143 (100.0%) | 104 (100.0%) |  |
| **E_histolytica** |  |  | - |  |  |  | - |
| No | 143 (100.0%) | 104 (100.0%) |  |  | 143 (100.0%) | 104 (100.0%) |  |
| **E_intestinalis** |  |  | 0.400 |  |  |  | 0.890 |
| Yes | 9 ( 6.3%) | 4 ( 3.8%) |  |  | 13 ( 9.1%) | 10 ( 9.6%) |  |
| No | 134 (93.7%) | 100 (96.2%) |  |  | 130 (90.9%) | 94 (90.4%) |  |
| **Isospora** |  |  | 0.820 |  |  |  | - |
| Yes | 1 ( 0.7%) | 1 ( 1.0%) |  |  | 0 ( 0.0%) | 0 ( 0.0%) |  |
| No | 142 (99.3%) | 103 (99.0%) |  |  | 143 (100.0%) | 104 (100.0%) |  |
| **Giardia** |  |  | 0.690 |  |  |  | 0.740 |
| Yes | 71 (49.7%) | 49 (47.1%) |  |  | 95 (66.4%) | 67 (64.4%) |  |
| No | 72 (50.3%) | 55 (52.9%) |  |  | 48 (33.6%) | 37 (35.6%) |  |
| **Cryptosporidium** |  |  | 0.830 |  |  |  | 0.360 |
| Yes | 22 (15.4%) | 15 (14.4%) |  |  | 30 (21.0%) | 27 (26.0%) |  |
| No | 121 (84.6%) | 89 (85.6%) |  |  | 113 (79.0%) | 77 (74.0%) |  |
| <= 35.00 = Yes |  |  |  |  |  |  |  |
| > 35.00 = No |  |  |  |  |  |  |  |
